# Comparison of foreign language anxiety based on four language skills in Chinese college students

**DOI:** 10.1186/s12888-022-04201-w

**Published:** 2022-08-19

**Authors:** Chao Ran, Yan Wang, Wan Zhu

**Affiliations:** 1 Institute of Foreign Languages, China Medical University, No77 Puhe Road, Shenyang North New Area, Shenyang, 110122 Liaoning Province China; 2 Department of Biomedical Engineering, School of Intelligent Medicine, China Medical University, Shenyang, China; 3 Department of Nosocomial Infection Control, The First Hospital of China Medical University, Shenyang, 110001 Liaoning China

**Keywords:** Foreign language anxiety, Language skills, College students

## Abstract

**Background:**

Numerous studies have established that foreign language anxiety (FLA) has a significant impact on learners’ language acquisition and performance. FLA is a unique form of anxiety that occurs in response to a certain circumstance. Even though a growing amount of research has extended to the examination of skill-based anxiety in specific, such as listening, speaking, reading, and writing, little used corresponding skilled-based FLA scales to assess learners’ skill-based FLA at one group of learners. To fill a void in this line of research, the study aimed to explore learners’ primary FLA by comparing their four language skill-specific FLAs with four different skill-based FLA scales. Additionally, we wished to investigate the variables that influence learners’ FLA.

**Methods:**

All participants in the study were first-year medical students. Individual instructors delivered and collected a total of 1023 questionnaires using an open questionnaire platform during normal English lessons in the mid-semester. SPSS 24.0 (Statistical Package for Social Science 24 version) was used to analyze all statistics. Internal validity tests were undertaken on each scale to ascertain the component structure of certain modified scales. The study employed the independent sample t-test and a statistical description to investigate students’ major FLA and its variables.

**Results:**

With a mean value of 106.863, the predominant FLA arouses from English listening anxiety. English reading anxiety was the lowest, with a mean score of 62.726. Male and female students both demonstrated the greatest degree of FLA in English listening and the least anxiety in English reading. However, their mean difference was not statistically significant (t = 1.220). By comparing the mean scores for four skill-based FLAs across language proficiency groups, it became clear that the scores for the medium were much higher than the average, with average scores of ESA: 91.988, ELA: 106.864, EWA: 74.157, ERA: 62.726, respectively, and the higher-level group scored lower than the average. Students’ prior English learning achievements are negatively connected with their FLA, with r values of −.207 (ELA), −.143 (EWA), and − .204 (ERA). The self-evaluation of students’ English listening, writing, and reading abilities was considerably adversely connected with FLA, but the self-evaluation of their English-speaking abilities was favorably correlated with FLA.

**Conclusion:**

A comparison of students’ FLA revealed that the primary skill-based FLA is related to English listening anxiety. Regardless of gender or language proficiency level, FLA was mainly driven by foreign language listening anxiety in all individuals. Prior language achievement and students’ self-evaluation are associated with their FLA.

**Supplementary Information:**

The online version contains supplementary material available at 10.1186/s12888-022-04201-w.

## Background

In foreign language classes, some students may display dry throat [1], clammy hands, tiredness [2], the avoidance of direct eye contact with their teachers [3, 4], and the avoidance of interactions during class [5]. All these negative behaviors and symptoms shown in the foreign language class are physical signs of Foreign Language Anxiety (FLA)—the concept introduced by Horwitz et al. in 1986. For decades, FLA has been a major topic in foreign language studies and has sparked several heated disputes.

FLA can be characterized as “a distinct complex of self-perceptions, beliefs, and behaviors related to classroom language learning arising from the uniqueness of the language learning process” [6]. A great amount of research has shown that FLA has a negative influence on students’ foreign language achievements or performance [4, 7]. Attempting to present an in-depth understanding of FLA, many individual variables and affective variables have been investigated, such as gender [8–11], age [12], FL proficiency level [13–15], the former experience of learning the target language [12, 16, 17], beliefs [18], motivation [19] and self-perception [20], etc.

In 1986, Horwitz introduced FLA as situation-specific anxiety aroused by a specific type of situation or event [21]. Suffering from situation-specific anxiety, individuals may consider specific events as anxiety-producing so long as certain factors are present. For instance, a person who could read English-language literature without experiencing any worry may experience severe anxiety when being required to speak in English publicly. Hence, every learner may experience different degrees of anxiety when acquiring four foreign language skills. Along with a continued interest in foreign/second language anxiety in general, a growing amount of research has extended to the examination of skill-based anxiety in specific, such as speaking anxiety [22–24], listening anxiety [7, 25, 26], reading anxiety [10, 27, 28], and writing anxiety [29, 30]. The majority of studies typically have centered upon the examination of one of the skill-based second/foreign language anxieties and its effects on language acquisition or performance. Findings have generally shown the independent existence of each of the four skill-based FLA and the negative relationships between one of the skill-based FLA and learners’ performance. Among them, there are some studies situated in all four skill-based FLA at once. For instance, Pea detected an independent existence of each of the four skill-based anxieties and each of them made a unique contribution to the prediction of FL classroom anxiety [31]. Abbaszadeh and Vizayaletchumi found a relationship existed between skill-based anxieties and language learning aptitude [32]. Luo et al. assessed heritage learners’ FLA based on all four language skills [33]. Torres and Turner investigated differences in students’ foreign language anxiety and foreign language self-efficacy related to skill-specific foreign language tasks (i.e., speaking, listening, reading, and writing) [34]. Piniel and Albert used qualitative research to present advanced learners’ foreign language-related emotional experiences across the four skills [35]. Jee examined the FLA and foreign language (FL) self-efficacy concerning four language skills [36].

In order to measure learners’ FLA, researchers created and used different skill-specific instruments or scales. For example, Horwitz’s Foreign Language Classroom Anxiety Scale (FLCAS) [37, 38]. Woodrow’s Second Language Speaking Anxiety Scale (SLSAS) [22]; Pae’s Speaking Anxiety Scale [31]; Foreign Language Listening Anxiety Scale (FLLAS) designed by Kim [7]; Foreign Language Reading Anxiety Scale (FLRAS) developed by Saito, Horwitz, & Garza [27] and Writing Anxiety Scale by Cheng [30]. So, as for the instruments used in the skill-based FLA, some studies put more importance on the development and validation of second language (L2) skill-specific anxiety scales [39]. Some studies used one scale to assess learners’ skill-based FLA [33, 34]. Some studies used different four skill-based scales to elicit learners’ four language skills [31, 32, 36].

The identification of skill-based anxiety enabled researchers to differentiate the role of language anxiety in learning different foreign/second language skills and represented learners’ foreign/second language anxiety profiles. However, it should be noted that regardless of the rich insights into skill-based FLA, the primary source of FLA based on four language skills in one group of students remain unanswered. Thus, the present study was intended to identify which of the foreign language skills elicits the highest level of learners’ FLA. The results of the study provide both statistical evidence of FLA and the contributing factor for FLA, which will contribute new knowledge to the existing study of FLA as well as the field of foreign language acquisition. Moreover, the findings will aid foreign language teachers in fostering a stress-free classroom atmosphere.

## Methodology

The main objective of this study is to conduct a cross-sectional study involving four skill-based FLAs of Chinese college students. Because the participants in this study were taught English as a foreign language, the term FLA relates to English anxiety in this study. The study addresses the following research questions:

1. Which is the primary FLA based on four language skills: speaking, listening, English reading, and writing?
2. What are the FLA differences between genders and varying degrees of language proficiency?
3. Is there any correlation between students’ prior foreign language achievements and their FLAs?

### Participants

The participants in this study were all first-year students at a medical university in a Chinese provincial capital city. The study included a total of approximately 300 participants; however, the number of final valid surveys for each scale varied. The general information of participants is depicted in Table 1. The participants’ general information is depicted in Table 1. All individuals had studied English in a regular classroom setting for at least 6 years and had completed the Chinese National College Entrance Examination before participating in this study. As a part of the general education requirements, all participants received a diverse English curriculum, which includes specialist courses in English listening and English speaking, intensive reading, academic English for medicine, and so forth. Each of them is required to pass National College English Test Band Four or Band Six to earn a bachelor’s degree. The language proficiency levels assigned to them were determined by their English scores on the Chinese National College Entrance Examination.

**Table 1:** Participants’ general information

	N			Frequency	Percentage	Range	Min	Max	Mean	Std. deviation
ESA	254	Gender	Male	74	29.1					
			Female	180	70.9					
		Age				7	16	23	18.29	.771
		Type of hometown	City	158	58.3					
			Town	54	21.2					
			Countryside	52	20.5					
		Years of studying English				9	3	12	9.74	2.258
ELA	249	Gender	Male	69	27.7					
			Female	180	72.3					
		Age				7	16	23	18.25	.789
		Type of hometown	City	145	58.2					
			Town	52	20.9					
			Countryside	52	20.9					
		Years of studying English				9	3	12	9.80	2.242
ERA	252	Gender	Male	67	26.6					
			Female	185	73.4					
		Age				7	16	23	18.26	.779
		Type of hometown	City	150	59.5					
			Town	50	19.8					
			Countryside	52	20.7					
		Years of studying English				10	3	12	9.79	2.203
EWA	268	Gender	Male	75	28					
			Female	193	72					
		Age				7	16	23	18.29	.802
		Type of hometown	City	159	59.3					
			Town	53	19.8					
			Countryside	56	20.9					
		Years of studying English				9	3	12	9.71	2.280

Note: *ESA* English Speaking Anxiety, *ELA* English Listening Anxiety, *ERA* English Reading Anxiety, *EWA* English Writing Anxiety

### Instrumentation

To ensure that participants understood the questions accurately and responded appropriately, a battery of scales prepared in Chinese was used in this study, including the English Listening Anxiety Scale (ELAS), English Speaking Anxiety Scale (ESAS), English Reading Anxiety Scale (ERAS), and English Writing Anxiety Scale (EWAS). Furthermore, appropriate modifications to the original scales were made to make them more applicable to the language learning circumstances of Chinese students. Specifically, the term “second language/foreign language” has been replaced with “English”. All scales consisted of two parts: the first part was the personal information of the participants, such as their age, gender, academic major, and self-evaluation; the second part contained questions designed to elicit students’ FLA. All scales were graded using a 5-point Likert scale. The answer continuum is as follows: 1 = Strongly Disagree, 2 = Disagree, 3 = Neither Agree nor Disagree, 4 = Agree, 5 = Strongly Agree. The greater the total score, the more severe the FLA. All items with negative characteristics were reversely rated. SPSS 24.0 (Statistical Package for Social Science 24 version) was used to statistically analyze the data. Internal validity tests were undertaken on each scale to evaluate the component structure of certain modified scales.

#### Measurement of English listening anxiety (ELA)

As Kim’s Foreign Language Listening Anxiety Scale (FLLAS) [7] has demonstrated an adequate degree of reliability and validity in previous studies, this study assessed participants’ English listening anxiety by adapting Kim’s FLLAS (see Additional file 1). Cronbach’s alpha reliability was.959 (*n* = 31), which is a significantly reliable value. Adapting FLLAS, Zhai stated a significant negative correlation between FLA and listening comprehension and put forward some useful suggestions to enhance the listening proficiency of foreign language learners [40].

#### Measurement of English-speaking anxiety (ESA)

The Foreign Language Classroom Anxiety Scale (FLCAS) [6] has been frequently used to assess foreign language speaking anxiety [29, 41]. However, foreign language speaking anxiety is distinct from overall FLA. As a result, using the FLCAS to assess learners’ foreign language speaking anxiety is inadequate. As a result, using the FLCAS to assess learners’ foreign language speaking anxiety is inadequate. Wu designed the Foreign Language Speaking Anxiety Self-Schema Questionnaire (FLSASQ) from the perspective of learners’ cognitive processing and the factors behind learners’ speaking anxiety [42]. Hence, the English-Speaking Anxiety Scale (ESAS) employed in this study was adapted from Wu’s FLSASQ (see Additional file 2). ESAS’s Cronbach’s alpha coefficient is 0.912. Rui and Ji explored college students’ speaking anxiety and classroom silence in China and found that Wu’s FLSASQ can explain the phenomenon of silence in the classroom very well [43].

#### Measurement of English Reading anxiety (ERA)

The English Reading Anxiety Scale (ERAS) was adapted from Saito’s Foreign Language Reading Anxiety Scale [27] to measure students’ anxiety levels when reading in English (see Additional file 3). ERAS’ Cronbach’s alpha coefficient stands at 0.84. That is, this scale has a high degree of internal validity. Akira Hamandaa and Shuichi Takaki examined the effects of multidimensional foreign language reading anxiety on achievement in Japanese English for foreign language (EFL) classrooms by using the FLRAS (Saito et al., 1999) [44].

#### Measurement of English writing anxiety (EWA)

EWAS (see Additional file 4) was used in this study to measure students’ foreign language writing anxiety. It was modified from Cheng’s [30] revised Second Language Writing Anxiety Inventory (SLWAI). Other researchers also tend to refer to Cheng’s SLWAI [45, 46]. The Cronbach’s alpha coefficient is equal to.891. Cheng et al. adopted FLWAS to explore the relationships between general L2 classroom anxiety and more skill-specific L2 writing anxiety [29].

### Procedure

Individual instructors disseminated and collected questionnaires during normal English lessons in mid-semester using an open questionnaire platform called Wenjuanxing. The questionnaire was accessible by participants via a link or a QR code presented in QQ—China’s social media group’s chatroom. Each questionnaire took approximately 10-15 minutes to complete.

### Data analysis

A total of 1023 questionnaires were collected. Among them, 254 represented ESAS, 249 represented ELAS, 268 represented EWAS, and 252 represented ERAS. SPSS V24.0 was used to analyze all these questionnaires (284 for males and 739 for females). First, descriptive statistics were constructed to compare the mean scores of students’ FLA. The participants were then classified according to their gender and language proficiency to provide a broad picture of the variances in students’ FLA. Third, the General Linear Model (GLM) approach was used to determine whether students’ prior English achievements and self-evaluation had any influence on FLA.

## Results

After the collection and analysis of the data from FLA scales through SPSS 24.0, the participants’ scores could be obtained.

### Statistics of FLAs in four scales

The ESAS includes 30 items, with a theoretical range of 30 to 150 scores for each student’s speaking anxiety. The ELAS consists of 31 items and yields a score between 31 and 155 points for each student’s listening anxiety. Since the EWAS has 25 items, the overall score for each student’s writing anxiety is likely to fluctuate between 25 and 125 points. Owing to the 20 questions in the ERAS, each student’s reading anxiety score may vary from 20 to 100. The descriptive analysis of the four FLA scales is shown in Table 2.

**Table 2:** Descriptive analysis of four skill-based FLAs

	N	Range	Min	Max	Mean	Std.deviation
ESA	254	120.00	30.00	150.00	91.988	9.197
ELA	249	117.00	38.00	155.00	106.863	21.074
EWA	268	79.00	41.00	120.00	74.157	13.793
ERA	252	60.00	40.00	100.00	62.726	10.461

Valid individual data were examined using SPSS 24.0. When the mean scores for four FLAs were compared, we found that students experienced more anxiety when listening to English than when speaking, writing, or reading it. That is, students demonstrated the highest degree of FLA in English listening, with scores ranging from 38 (minimum) to 155 (maximum), with a range of 117. According to Table 2, the total mean score for ELA was 106.863, which was quite high among the four FLAs. It was revealed that students were substantially worried when listening to English in and out of class. In addition, 49% of participants were found to be below the mean, while 51% were found to be above the mean. Moreover, the mean score of ERA was 62.726, which was the lowest of the four FLAs, followed by ESA (91.988) and EWA (74.157). Based on the total assessment of these four FLAs, we had a clearer view of the participants’ primary FLA. It was necessary to dig deeper into the specific and comprehensive explanation of the four skill-based FLAs.

### Gender differences in FLA

A significant number of studies have found a relationship between gender and FLA. For instance, Dewaele et.al. revealed that female learners reported less FLA than male learners in their foreign language classroom [47]. Other studies found that female learners experienced more FLA than their male counterparts [48, 49]. Surprisingly, Dewaele et al. found that male Kazakh learners of Turkish experienced higher levels of FLA in the classroom than their female peers [50]. Some studies have found no gender differences [51]. Even if these gender-related studies yielded rather conflicting results, it has been widely suggested that males and females identify and react differently to evaluative situations. An independent sample t-test of gender difference was used to identify students’ ESA, ELA, EWA, and ERA. Male and female students both had the largest degree of FLA in listening and the lowest degree of anxiety FLA in reading, as shown in Table 3. A further investigation indicated that while male students in ELA (M = 109.529) reported higher degrees of anxiety than female students (M = 105.867), the difference was not statistically significant (t = 1.220).

**Table 3:** FLA differences in gender

	Gender	N	Mean	Std.deviation
ESA	male	74	91.622	11.315
	female	180	92.139	8.201
ELA	male	68	109.529	22.591
	female	180	105.867	20.508
EWA	male	75	75.867	16.070
	female	193	73.492	12.785
ERA	male	67	63.851	10.810
	female	185	62.319	10.332

Therefore, even though male students experienced greater levels of anxiety in ELA than female students, their disparities in FLA did not reach statistical significance. Overall, male students consistently displayed higher anxiety than female students in all aspects of English, and the variations between male and female students were fewer, i.e., There were no statistically significant differences between the two groups.

### Anxiety differences in different language proficiency groups

There were 234 students in the ESA session, with 171 students in the advanced group, 62 students in the medium group, and 1 student in the lower group. There were 229 students in the ELA session, including 166 students in the advanced group, 61 participants in the medium group, and 2 students in the lower group. EWA had a total of 251 students, consisting of 180 students in the advanced group, 68 students in the medium group, and three students in the lower group. ERA had 171 students in the advanced group, 66 students in the medium group, and 2 students in the lower group, making up the total of 239 students enrolled. A comparison of individuals in different groups with their differing FLAs is shown in Table 4.

**Table 4:** FLA differences in different language proficiency groups

		N	Range	Min	Max	Mean	Std.deviation
ESA	Advanced	171	120.00	30.00	150.00	91.883	10.128
	Medium	62	31.00	77.00	108.00	92.597	6.535
ELA	Advanced	166	117.00	38.00	155.00	103.994	21.163
	Medium	61	89.00	66.00	155.00	112.410	19.426
EWA	Advanced	180	79.00	41.00	120.00	73.206	14.298
	Medium	68	66.00	50.00	116.00	76.588	12.300
ERA	Advanced	171	60.00	40.00	100.00	61.462	10.424
	Medium	66	46.00	40.00	86.00	65.652	10.087

Note: The number of participants in the lower group is extremely less (1 participant in ESA; 2 participants in ELA; 3 participants in EWA; 2 participants in ERA). The report of the participants in the lower group is insignificant regarding all forms of anxiety. So, the report of participants in the lower group in Table 4 and their data analysis was removed

According to a comparison of mean scores of ESA, ELA, EWA, and ERA, the greatest FLA experienced by students with varied language levels came from English listening. Table 4 clearly shows that the scores of the medium group were higher than the average, with average scores of ESA: 91.988, ELA: 106.864, EWA: 74.157, and ERA: 62.726, respectively, and the score of the advanced group was below the average score. It recommended that students with high language capacities be less anxious than those with medium capacities.

The descriptive analysis of four skill-based FLAs for various language proficiency groups also revealed that there were relatively significant differences between the advanced and medium language proficiency groups. That is, the difference between the advanced and medium proficiency language groups was large, especially in ELA, where the mean value was 103.994 for the advanced group and the value of the mean was 112.410 for the medium group. Considering the four skill-based FLA, ELA had the highest difference value among them, with a different value of 8.416, followed by ERA, with a difference of 5.19, and a difference value of EWA of 3.382. A less significant difference in speaking anxiety existed between the advanced group and the medium group, with a difference of 0.714. Furthermore, with regard to ESA, the differences within the medium group were extremely small, with a range of 31.00 and a standard deviation of 6.535. Regarding the ELA, the differences within the advanced group are significantly large, with a range of 117.00 and a standard deviation of 21.163. The FLA scores for the two groups were negatively correlated with their language proficiency levels, namely, the group with better language skills had less FLA and vice versa.

Even though advanced students perceived the lowest FLA compared with medium group of students, there were still different presentations of FLA in the advanced group. It was clear from looking at the range and standard deviation values presented in Table 4 that there was a substantial difference in English-speaking anxiety for the advanced group, with a range of 120 and a standard deviation of 10.128. The results indicated that English-speaking anxiety appeared to be extremely high in the advanced group; that is, even with a high speaking ability, some students maintain a relatively high level of anxiety while speaking English, while others maintained less anxiety about their performance. Most likely, despite their strong language skills, they had multiple negative self-evaluations, lacked confidence, or were scared of failure, all of which imposed a severe burden on them.

### Analysis of the influence of prior foreign language achievement on students’ FLA

Considering that FAL is a psychological concept, experience or accomplishment should influence learners’ language acquisition and performance. There have been many studies that have confirmed the association between FLA and foreign language achievement in the field of FLA [37, 52]. That is, a learner with a significant level of FLA achieves low levels of proficiency in foreign language acquisition. However, it is still unknown whether there is a correlation between learners’ previous foreign language performance and their FLA. As a result, this study examined the correlation between FLA and learners’ prior foreign language achievement using learners’ English scores on the National College Entrance Exam as a variable.

As seen in Table 5, students’ prior English learning achievements were adversely connected with their FLA, as indicated by the r of −.207(ELA), −.143(EWA), and − .204(ERA). It was anticipated that students with lower prior foreign language achievement in English learning would have higher FLA, whereas those with higher prior achievement in English learning would experience lower FLA. Surprisingly, there was no statistical correlation between students’ ESA and prior foreign language achievement. Further investigation revealed that students’ prior English learning achievements had a reasonably strong negative association with ELA, with an r value of −.207.

**Table 5:** Correlation analysis between FLA and participants’ prior foreign language achievement

		ESA	ELA	EWA	ERA
PAEL	Pearson correlation	−.062	−.207^**^	−.143^*^	−.204^**^
	Sig. (2-tailed)	.344	.002	.023	.002
	N	234	229	251	239

Note. ^*^ *P <* .05; ^**^P < .01

Note: *PAEL* The previous achievement in English learning

### Analysis of the influence of student’s self-evaluation on FLA

As a process of self-analysis, self-evaluation has been identified as a critical feature in FLA in multiple studies [53]. Thus, self-evaluation should be considered to have a holistic view of learners’ FLA.

From Table 6, it could be seen that students’ self-evaluation of their four English skills affected their degree of FLA. Table 6 also demonstrated that self-evaluation of English listening, English writing, and English reading was significantly negatively correlated with FLA. That is students who preferred English listening, English writing, and English reading and had a high self-evaluation of these abilities perceived less FLA, and vice versa. However, it was shown that students’ self-evaluation was positively connected with their level of speaking anxiety. This is probably because anxious students frequently overestimate their real language proficiency [54].

**Table 6:** Correlation analysis of self-evaluation on four English skills

		Self-evaluation	Anxiety scores
Self-evaluation of English listening	Pearson correlation	1	−.490^**^
	Sig. (2-tailed)		.000
	N	249	249
Self-evaluation of English speaking	Pearson correlation	1	.346^**^
	Sig. (2-tailed)		.000
	N	254	254
Self-evaluation of English writing	Pearson correlation	1	−.294**
	Sig. (2-tailed)		.000
	N	268	268
Self-evaluation of English reading	Pearson correlation	1	−.361^**^
	Sig. (2-tailed)		.000
	N	252	252

Note. * *P<*.05; **P<.01

## Discussion

This study is to horizontally explore the degree of FLA among Chinese college students considering four foreign language skills: listening, reading, speaking, and writing. The study’s findings demonstrated that students experience or display their FLA in the following sequence: listening, speaking, writing, and reading. In other words, FLA has a greater impact on the listening and speaking abilities of foreign language students than on their writing and reading abilities. This means that speaking and listening are two significant sources of FLA [4]. The results were partly in agreement with the findings of Bilá [55] and Horwitz et al. [6]. In their studies, foreign language learners reported strong speaking anxiety and indicated their inadequate speaking ability as the strongest barrier in foreign language communication. This finding provided supplementary evidence for the primary origins of FLA related to four foreign language skills. Additionally, it has been shown that foreign language students express less anxiety about their foreign language reading than they do about their foreign language speaking [56], which may result from different input and output forms. In terms of foreign language-specific abilities, listening and speaking may be viewed as forms of auditory input and output, whereas writing and listening can be viewed as types of visual input and output. Foreign language listening is a difficult cognitive activity [57] that requires the processing of a variety of aural inputs. Therefore, the listening process places a high demand on cognitive resources such as attention and memory [58]. FLA has been shown to hinder attention and memory [59]. The distraction of attention or obstruction of memory might reduce the quantity of processed auditory information [59]. Thus, FLA can have a significant detrimental influence on listening performance. In this study, 59% of students indicated that they were concerned about missing critical information as a result of attentional distraction. Likewise, other major sources of listening anxiety were the characteristics of linguistic input. A total of 54.6% of students in this study expressed concern about not comprehending what the speaker is saying owing to the speaker’s rapid pace of speaking. When students are unfamiliar with the speakers’ tone, 41.4% of students experience FLA. The possible reason may be that students are unable to constantly stop a conversation to request explanations or ask for repetition, which increases their degree of FLA. On the other hand, FLA had the fewest adverse effects on foreign language reading; that is, FLA contributes far less to visual input processing. Since the degree of ERA was the lowest anxiety among the four FLAs, foreign language reading may be a less unpleasant task for foreign language students. Given that foreign reading is a less demanding activity and causes less anxiety, it is unsurprising that foreign language reading anxiety has a weaker link with reading performance. In contrast to auditory input, which is often given sequentially, reading frequently enables students to read the material at their own pace and have the flexibility of looking up information in any paragraph of a text to help in comprehension [60], even with the assistance of additional resources. Given this fact, it is possible to explain why anxious students struggled to acquire oral skills (listening and speaking).

Regarding FLA differences in genders and language proficiency levels, the results indicated that gender differences in FLA were not significant, although male students experienced slightly higher FLA than female students. This may be explained by poor self-esteem for foreign language proficiency [16]. As predicted, the findings corroborated those found in Kitano’s investigations [16]. However, this study contradicted with what Boudreau & Dewaele’s study. They discovered that female learners had a significantly higher degree of FLA than their male peers [47].

Concerning the FLA at various degrees of language proficiency, the finding demonstrates that the degree of FLA is related to foreign language proficiency: students with high foreign language proficiency often express less anxiety than starting learners. That is, the stronger learners’ foreign language proficiency is, the less anxiety they perceive [26, 61]. Students with better language competence expressed greater confidence in their foreign language performance, while students with poorer language proficiency expressed lower confidence. The findings were in line with the results of Jee and Ewald’s study [13, 14], and echoed Sparks & Ganschow’s claim that “language skills are likely to be a confounding variable … anxiety plays a primary role in foreign language proficiency and achievement” [15]. Furthermore, regardless of students’ language proficiency levels, their primary source of FLA was typically foreign language listening. Although the degree of FLA may change as a function of proficiency [15, 26], highly proficient learners can also demonstrate a high degree of FLA while speaking a foreign language [62]. Thus, these findings added to the evidence that students’ foreign language proficiency levels did not influence their anxiety over foreign language listening.

Regarding the influence of prior foreign language achievement on students’ FLA, this study revealed that students with strong prior English learning achievements perceived a lower FLA than students with poor prior English learning achievements. The findings were consistent with the finding of Onwuegbuzie et al.. They found that prior high school experience determined the level of students’ FLA [12]. Hence, this research adds to the evidence that FLA is associated with both prior and posterior foreign-language performance.

When FLA was correlated with students’ self-evaluation, it was shown in this study that students who are very typically self-evaluative report having a lower degree of FLA in listening, writing, and reading. This discovery corroborated the findings of Bailey et al., Yamini et al., and Young [63–65]. It also verified the claim made by Kitano that “individual student’s anxiety was higher as he or she perceived his or her ability as lower than that of peers and native speakers” [16]. Highly anxious students have a low evaluation of their academic ability and self-worth [12]. However, the study found that students who place a high premium on English speaking earn higher FLA scores, whereas those who place a low premium on English speaking earn lower FLA scores. That is, students with a strong sense of self-evaluation typically experienced a greater degree of FLA in their ability to communicate.

## Conclusion

This study has provided evidence for discussing FLA in terms of foreign language-specific skills. The study showed that the primary cause of FLA is listening anxiety related to four language skills. Students, regardless of their language proficiency level or gender, are all anxious about foreign language listening. In this respect, teachers must sensitize themselves to students’ emotional reactions, such as facial expressions, gestures, and voices in classroom interactions [66]. Accordingly, specific teaching strategies may be employed to make the negative effects of FLA benefit students’ FL learning, especially FLLA. This study also demonstrated that students’ foreign language achievement and self-evaluation are inextricably correlated with FLA. In this respect, teachers can offer positive English learning opportunities [67] and constantly compliment even small progress in English learning [20] to accumulate students’ confidence and successful experiences.

Nonetheless, the present study’s findings should be interpreted in light of various limitations. First, since it was performed with a restricted group of volunteers, caution should be maintained regarding the generalizability of the results. Second, this study does not go into further detail on why self-elevation correlates differently with four language skills. Last, since FLA is a dynamic psychological phenomenon instead of a static one, longitudinal research utilizing a variety of approaches is necessary.

Future studies of FLA may employ qualitative instruments, such as interview classroom observations, or teacher reflections to enrich the knowledge of skill-based FLA. Moreover, other affective variables such as learner’s L1, motivation, personality, and hometown cultural differences are also worth investigating.

## Supplementary Information

**Additional file 1.**

**Additional file 2.**

**Additional file 3.**

**Additional file 4.**

## Data Availability

The datasets used and/or analyzed during the current study are not publicly available due we signed confidentiality agreements with the research participants but are available from the corresponding author upon reasonable request.

## Abbreviations

FLA: Foreign Language Anxiety
FL: Foreign language
FLCAS: Foreign Language Classroom Anxiety Scale
SLSAS: Second Language Speaking Anxiety Scale
FLLAS: Foreign Language Listening Anxiety Scale
FLRAS: Foreign Language Reading Anxiety Scale
L2: Second language
ESA: English Speaking Anxiety
ELA: English Listening Anxiety
ERA: English Reading Anxiety
EWA: English Writing Anxiety
ELAS: English Listening Anxiety Scale
ESAS: English-Speaking Anxiety Scale
ERAS: English Reading Anxiety Scale
EWAS: English Writing Anxiety Scale
SPSS 24.0: Statistical Package for Social Science 24 version
FLSASQ: Foreign Language Speaking Anxiety Self-Schema Questionnaire
EFL: English for foreign language
SLWAI: Second Language Writing Anxiety Inventory
GLM: General Linear Model
PAEL: The previous achievement in English learning
