# Supplementary material for: Comparison of foreign language anxiety based on four language skills in Chinese college students
Source: BMC Psychiatry. 2022 Aug 19;22:558. doi: 10.1186/s12888-022-04201-w (PMC9389700; doi:10.1186/s12888-022-04201-w)
Supplement: Supplementary file 1 — Additional file 1. [file 12888_2022_4201_MOESM1_ESM.pdf]

English Listening Anxiety Scale

Direction: All the questions in both Part A and Part B are for research purposes only, and any information obtained will remain confidential.

Part A:

Direction: Please answer the following questions or make a √ in the box next to the statement that fits your current situation.

- Sex: ____________
- Age： ____________years old
- Your hometown is a： □City □Town □Countryside
- How many years have you studied English?
  □Less than 3 years □6 years □9 years □More than 12 years
- English scores on the Chinese National College Entrance Examination ____________
- How would you rate your listening proficiency in English on a scale of one to five?_______
  □Poor □Not good □Moderate □Good □Excellent

Part B:

Direction: The following statements apply to how people feel in the process of listening to native speakers of English. Please, place a √ in the box next to the statement that fits your ideas.

1. When listening to English, I tend to get stuck on one or two unknown words.
   □Strongly disagree □Disagree □Neither Agree nor Disagree □Agree □Strongly agree
2. I get nervous if a listening passage is read only once during English listening tests.
   □Strongly disagree □Disagree □Neither Agree nor Disagree □Agree □Strongly agree
3. When a person speaks English very fast, I worry that I might not understand all of it.
   □Strongly disagree □Disagree □Neither Agree nor Disagree □Agree □Strongly agree
4. I am nervous when I am listening to English if 1 am not familiar with the topic.
   □Strongly disagree □Disagree □Neither Agree nor Disagree □Agree □Strongly agree
5. It’s easy to guess about the parts that I miss while listening to English.
   □Strongly disagree □Disagree □Neither Agree nor Disagree □Agree □Strongly agree
6. If I let my mind drift even a little bit while listening to English. I worry that I will miss important ideas.
   □Strongly disagree □Disagree □Neither Agree nor Disagree □Agree □Strongly agree
7. When I’m listening to English. I am worried when I can’t watch the lips or facial expression of a person who is speaking.
   □Strongly disagree □Disagree □Neither Agree nor Disagree □Agree □Strongly agree
8. During English listening tests. I get nervous and confused when I don’t understand every word.
   □Strongly disagree □Disagree □Neither Agree nor Disagree □Agree □Strongly agree
9. When listening to English, it is difficult to differentiate the words from one another.
   □Strongly disagree □Disagree □Neither Agree nor Disagree □Agree □Strongly agree
10. 1 feel uncomfortable in class when listening to English without the written text.
    □Strongly disagree □Disagree □Neither Agree nor Disagree □Agree □Strongly agree

11. When I’m listening to English, I often get so confused I can’t remember what I have heard.
□Strongly disagree □Disagree □Neither Agree nor Disagree □Agree □Strongly agree

12. My thoughts become jumbled and confused when listening to important information in English listening.
□Strongly disagree □Disagree □Neither Agree nor Disagree □Agree □Strongly agree

13. I get worried when I have little time to think about what I hear in English.
□Strongly disagree □Disagree □Neither Agree nor Disagree □Agree □Strongly agree

14. When I’m listening to English. I usually end up translating word by word without understanding the contents.
□Strongly disagree □Disagree □Neither Agree nor Disagree □Agree □Strongly agree

15. .1 would rather not have to listen to people speak English at all.
□Strongly disagree □Disagree □Neither Agree nor Disagree □Agree □Strongly agree

16. 1 get worried when I can’t listen to English at my own pace.
□Strongly disagree □Disagree □Neither Agree nor Disagree □Agree □Strongly agree

17. 1 keep thinking that everyone else except me understands very well what an English speaker is saying.
□Strongly disagree □Disagree □Neither Agree nor Disagree □Agree □Strongly agree

18. I get upset when I’m not sure whether I understand what I am listening to English.
□Strongly disagree □Disagree □Neither Agree nor Disagree □Agree □Strongly agree

19. If a person speaks English very quietly. I am worried about understanding.
□Strongly disagree □Disagree □Neither Agree nor Disagree □Agree □Strongly agree

20. It’s difficult for me to listen to English when there is even a little bit of background noise.
□Strongly disagree □Disagree □Neither Agree nor Disagree □Agree □Strongly agree

21. Listening to new information in English makes me uneasy.
□Strongly disagree □Disagree □Neither Agree nor Disagree □Agree □Strongly agree

22. 1 get annoyed when I come across words that I don’t understand while listening to English.
□Strongly disagree □Disagree □Neither Agree nor Disagree □Agree □Strongly agree

23. It is difficult for me to listen to English when English stress and intonation is the unfamiliar.
□Strongly disagree □Disagree □Neither Agree nor Disagree □Agree □Strongly agree

24. When listening to English. I often understand the words but still can’t quite understand what the speaker means.
□Strongly disagree □Disagree □Neither Agree nor Disagree □Agree □Strongly agree

25. It frightens me when I cannot catch a key word of an English listening passage.
□Strongly disagree □Disagree □Neither Agree nor Disagree □Agree □Strongly agree

26. I fear I have inadequate background knowledge of some topics when listening in English.
□Strongly disagree □Disagree □Neither Agree nor Disagree □Agree □Strongly agree

27. 1 have difficulty understanding oral instructions given to me in English.
□Strongly disagree □Disagree □Neither Agree nor Disagree □Agree □Strongly agree

28. It is hard to concentrate on what English speakers are saying unless I know them well.

□Strongly disagree □Disagree □Neither Agree nor Disagree □Agree □Strongly agree

29. I feel confident when I am listening in English.

□Strongly disagree □Disagree □Neither Agree nor Disagree □Agree □Strongly agree

30. 1 am nervous when listening to an English speaker on the phone or when imagining a situation where I listen to an English speaker on the phone.

□Strongly disagree □Disagree □Neither Agree nor Disagree □Agree □Strongly agree

31. 1 feel tense when listening to English as a member o f a social gathering or when imagining a situation where I listen to English as a member of a social gathering.

□Strongly disagree □Disagree □Neither Agree nor Disagree □Agree □Strongly agree
