# Supplementary material for: Comparison of foreign language anxiety based on four language skills in Chinese college students
Source: BMC Psychiatry. 2022 Aug 19;22:558. doi: 10.1186/s12888-022-04201-w (PMC9389700; doi:10.1186/s12888-022-04201-w)
Supplement: Supplementary file 2 — Additional file 2. [file 12888_2022_4201_MOESM2_ESM.pdf]

English Speaking Anxiety Scale(ESAS)

Direction: All the questions in both Part A and Part B are for research purposes only, and any information obtained will remain confidential.

Part A:

Direction: Please answer the following questions or make a √ in the box next to the statement that fits your current situation.

- Sex: ____________
- Age： ____________years old
- Your hometown is a： □City □Town □Countryside
- How many years have you studied English?

  □Less than 3 years □6 years □9 years □More than 12 years
- English scores on the Chinese National College Entrance Examination ____________
- How would you rate your English-speaking proficiency in English on a scale of one to five?_______

  □Poor □Not good □Moderate □Good □Excellent

Part B:

Direction: The following statements apply to how people feel in the process of listening to native speakers of English. Please, place a √ in the box next to the statement that fits your ideas.

1. If I work hard, I can be the best English speaker in my class.

   □Strongly disagree □Disagree □Neither Agree nor Disagree □Agree □Strongly agree
2. I am very confident in improving my English speaking.

   □Strongly disagree □Disagree □Neither Agree nor Disagree □Agree □Strongly agree
3. It is really difficult for me to talk with others in English.

   □Strongly disagree □Disagree □Neither Agree nor Disagree □Agree □Strongly agree
4. I feel that my English speaking is very poor.

   □Strongly disagree □Disagree □Neither Agree nor Disagree □Agree □Strongly agree
5. I'm talented in learning foreign languages.

   □Strongly disagree □Disagree □Neither Agree nor Disagree □Agree □Strongly agree
6. I'm a fast learner of English as a foreign language.

   □Strongly disagree □Disagree □Neither Agree nor Disagree □Agree □Strongly agree
7. Learning to speak in English is not easy.

   □Strongly disagree □Disagree □Neither Agree nor Disagree □Agree □Strongly agree
8. I can speak English fluently.

   □Strongly disagree □Disagree □Neither Agree nor Disagree □Agree □Strongly agree
9. I often wonder if I can learn to speak English well.

   □Strongly disagree □Disagree □Neither Agree nor Disagree □Agree □Strongly agree
10. In English class, I don't worry about making mistakes when I speak in English.

    □Strongly disagree □Disagree □Neither Agree nor Disagree □Agree □Strongly agree
11. In English class, I always make the worst prediction when English teacher will make comment on my answers.

□Strongly disagree □Disagree □Neither Agree nor Disagree □Agree □Strongly agree

12. When speaking in English, I am afraid of looking stupid.

□Strongly disagree □Disagree □Neither Agree nor Disagree □Agree □Strongly agree

13. When speaking in English, I was worried that other students would make fun of me.

□Strongly disagree □Disagree □Neither Agree nor Disagree □Agree □Strongly agree

14. When speaking English, I don't care what people think of me.

□Strongly disagree □Disagree □Neither Agree nor Disagree □Agree □Strongly agree

15. I often reflect on my process of learning spoken English.

□Strongly disagree □Disagree □Neither Agree nor Disagree □Agree □Strongly agree

16. My classmates are envious of my spoken English.

□Strongly disagree □Disagree □Neither Agree nor Disagree □Agree □Strongly agree

17. I feel a sense of achievement in my spoken English.

□Strongly disagree □Disagree □Neither Agree nor Disagree □Agree □Strongly agree

18. I feel very confident when I speak in English class.

□Strongly disagree □Disagree □Neither Agree nor Disagree □Agree □Strongly agree

19. I will panic when making an unprepared speech in English class.

□Strongly disagree □Disagree □Neither Agree nor Disagree □Agree □Strongly agree

20. I felt my heart beat faster when I was called on by the teacher in English class.

□Strongly disagree □Disagree □Neither Agree nor Disagree □Agree □Strongly agree

21. I don't get nervous talking to the teacher in English class.

□Strongly disagree □Disagree □Neither Agree nor Disagree □Agree □Strongly agree

22. I would like to spend extra time practicing my spoken English.

□Strongly disagree □Disagree □Neither Agree nor Disagree □Agree □Strongly agree

23. I always try to improve my English-speaking skills.

□Strongly disagree □Disagree □Neither Agree nor Disagree □Agree □Strongly agree

24. When I don't know how to speak in English, I ask my teacher or classmates for help.

□Strongly disagree □Disagree □Neither Agree nor Disagree □Agree □Strongly agree

25. I don't want to learn to speak English.

□Strongly disagree □Disagree □Neither Agree nor Disagree □Agree □Strongly agree

26. In English class, I often think about irrelevant things.

□Strongly disagree □Disagree □Neither Agree nor Disagree □Agree □Strongly agree

27. Usually, I am not active in reading English outside of class.

□Strongly disagree □Disagree □Neither Agree nor Disagree □Agree □Strongly agree

28. In English class, I like to sit in the corner of the classroom.

□Strongly disagree □Disagree □Neither Agree nor Disagree □Agree □Strongly agree

29. I often don't want to go to English classes.

□Strongly disagree □Disagree □Neither Agree nor Disagree □Agree □Strongly agree
