# Supplementary material for: Comparison of foreign language anxiety based on four language skills in Chinese college students
Source: BMC Psychiatry. 2022 Aug 19;22:558. doi: 10.1186/s12888-022-04201-w (PMC9389700; doi:10.1186/s12888-022-04201-w)
Supplement: Supplementary file 3 — Additional file 3. [file 12888_2022_4201_MOESM3_ESM.pdf]

English Reading Anxiety Scale (ERAS)

Direction: All the questions in both Part A and Part B are for research purposes only, and any information obtained will remain confidential.

Part A:

Direction: Please answer the following questions or make a √ in the box next to the statement that fits your current situation.

- Sex: ____________
- Age： ____________years old
- Your hometown is a： □City □Town □Countryside
- How many years have you studied English?

  □Less than 3 years □6 years □9 years □More than 12 years
- English scores on the Chinese National College Entrance Examination ____________
- How would you rate your English reading ability on a scale of one to five?_______

  □Poor □Not good □Moderate □Good □Excellent

Part B:

Direction: The following statements apply to how people feel in the process of listening to native speakers of English. Please, place a √ in the box next to the statement that fits your ideas.

1. I get upset when I'm not sure whether I understand what I am reading in English.

   □Strongly disagree □Disagree □Neither Agree nor Disagree □Agree □Strongly agree
2. When reading in English. I often understand the words but still can't quite understand what the writer means.

   □Strongly disagree □Disagree □Neither Agree nor Disagree □Agree □Strongly agree
3. When I'm reading English, I get so confused that I can't remember what I have read.

   □Strongly disagree □Disagree □Neither Agree nor Disagree □Agree □Strongly agree
4. 1 feel intimate whenever I see a whole page of English in front of me.

   □Strongly disagree □Disagree □Neither Agree nor Disagree □Agree □Strongly agree
5. I am nervous when I am reading a passage in English when I am not familiar with the topic.

   □Strongly disagree □Disagree □Neither Agree nor Disagree □Agree □Strongly agree
6. I get upset whenever I encounter unknown grammar when reading English.

   □Strongly disagree □Disagree □Neither Agree nor Disagree □Agree □Strongly agree
7. When reading English, I get nervous and confused when I don't understand every word.

   □Strongly disagree □Disagree □Neither Agree nor Disagree □Agree □Strongly agree
8. It bothers me to encounter words I can't pronounce correctly while reading English.

   □Strongly disagree □Disagree □Neither Agree nor Disagree □Agree □Strongly agree
9. I usually end up translating word by word without understanding the content when I'm reading English.

   □Strongly disagree □Disagree □Neither Agree nor Disagree □Agree □Strongly agree
10. When I came across funny letters and symbols in English, it's hard for me to remember what I have read.

    □Strongly disagree □Disagree □Neither Agree nor Disagree □Agree □Strongly agree

11. I am worried about all the new words I have to learn to read in English.

□Strongly disagree □Disagree □Neither Agree nor Disagree □Agree □Strongly agree

12. I enjoy reading in English.

□Strongly disagree □Disagree □Neither Agree nor Disagree □Agree □Strongly agree

13. I feel confident when I am reading in English.

□Strongly disagree □Disagree □Neither Agree nor Disagree □Agree □Strongly agree

14. Once I get used to it, reading English is not so difficult.

□Strongly disagree □Disagree □Neither Agree nor Disagree □Agree □Strongly agree

15. The hardest part of learning English is learn to read.

□Strongly disagree □Disagree □Neither Agree nor Disagree □Agree □Strongly agree

16. I would be happy to learn to speak in English rather than learning to read in English.

□Strongly disagree □Disagree □Neither Agree nor Disagree □Agree □Strongly agree

17. I don't mind reading to myself, but I feel very uncomfortable when I have to read English aloud.

□Strongly disagree □Disagree □Neither Agree nor Disagree □Agree □Strongly agree

18. I am satisfied with the English reading ability that I have achieved so far.

□Strongly disagree □Disagree □Neither Agree nor Disagree □Agree □Strongly agree

19. English culture seems very foreign to me.

□Strongly disagree □Disagree □Neither Agree nor Disagree □Agree □Strongly agree

20. I have to know about so much English history and culture to read English.

□Strongly disagree □Disagree □Neither Agree nor Disagree □Agree □Strongly agree
