# Supplementary material for: Comparison of foreign language anxiety based on four language skills in Chinese college students
Source: BMC Psychiatry. 2022 Aug 19;22:558. doi: 10.1186/s12888-022-04201-w (PMC9389700; doi:10.1186/s12888-022-04201-w)
Supplement: Supplementary file 4 — Additional file 4. [file 12888_2022_4201_MOESM4_ESM.pdf]

English Writing Anxiety Scale (EWAS)

Direction: All the questions in both Part A and Part B are for research purposes only, and any information obtained will remain confidential.

Part A:

Direction: Please answer the following questions or make a √ in the box next to the statement that fits your current situation.

- Sex: ____________
- Age： ____________years old
- Your hometown is a： □City □Town □Countryside
- How many years have you studied English?

  □Less than 3 years □6 years □9 years □More than 12 years
- English scores on the Chinese National College Entrance Examination ____________
- How would you rate your writing ability in English on a scale of one to five?_______

  □Poor □Not good □Moderate □Good □Excellent

Part B:

Direction: The following statements apply to how people feel in the process of listening to native speakers of English. Please, place a √ in the box next to the statement that fits your ideas.

1. I am not nervous at all when writing in English,.

   □Strongly disagree □Disagree □Neither Agree nor Disagree □Agree □Strongly agree
2. I feel my heart pounding when I write English compositions in a limited time.

   □Strongly disagree □Disagree □Neither Agree nor Disagree □Agree □Strongly agree
3. I am worried about being unable to use the tense and grammar correctly when writing an English composition.

   □Strongly disagree □Disagree □Neither Agree nor Disagree □Agree □Strongly agree
4. When I am writing an English composition, I am worried about not being able to write complex sentences.

   □Strongly disagree □Disagree □Neither Agree nor Disagree □Agree □Strongly agree
5. I fear that I can’t use English collocation correctly.

   □Strongly disagree □Disagree □Neither Agree nor Disagree □Agree □Strongly agree
6. I was worried about not being able to make my English writing more coherent and fluent.

   □Strongly disagree □Disagree □Neither Agree nor Disagree □Agree □Strongly agree
7. I feel upset if I’m unable to express my ideas clearly and understandably in my English writing.

   □Strongly disagree □Disagree □Neither Agree nor Disagree □Agree □Strongly agree
8. I feel worried and uneasy if I know my English writing will be evaluated.

   □Strongly disagree □Disagree □Neither Agree nor Disagree □Agree □Strongly agree
9. I often choose to write down my thoughts in English.

   □Strongly disagree □Disagree □Neither Agree nor Disagree □Agree □Strongly agree
10. My mind often goes blank when I start to write in English.

    □Strongly disagree □Disagree □Neither Agree nor Disagree □Agree □Strongly agree

11. I don’t think that my English writing is worse than others’.

□Strongly disagree □Disagree □Neither Agree nor Disagree □Agree □Strongly agree

12. If my English writing is to be evaluated, I would worry about getting a very poor grade.

□Strongly disagree □Disagree □Neither Agree nor Disagree □Agree □Strongly agree

13. I usually try to avoid writing in English.

□Strongly disagree □Disagree □Neither Agree nor Disagree □Agree □Strongly agree

14. My thoughts become jumbled when I write English compositions under heavy time pressure.

□Strongly disagree □Disagree □Neither Agree nor Disagree □Agree □Strongly agree

15. I would not write in English if I have choice.

□Strongly disagree □Disagree □Neither Agree nor Disagree □Agree □Strongly agree

16. I often feel panic when I write in English within a limited time.

□Strongly disagree □Disagree □Neither Agree nor Disagree □Agree □Strongly agree

17. It frightens me that the other students would deride my English writing if they read it.

□Strongly disagree □Disagree □Neither Agree nor Disagree □Agree □Strongly agree

18. I freeze up when being asked to write English compositions unexpectedly.

□Strongly disagree □Disagree □Neither Agree nor Disagree □Agree □Strongly agree

19. I would do my best to make an excuse not to write English writing.

□Strongly disagree □Disagree □Neither Agree nor Disagree □Agree □Strongly agree

20. I don’t worry at all about what other people would think of my English writing.

□Strongly disagree □Disagree □Neither Agree nor Disagree □Agree □Strongly agree

21. I usually seek every possible chance to write in English outside of class.

□Strongly disagree □Disagree □Neither Agree nor Disagree □Agree □Strongly agree

22. I usually feel my whole body rigid and tense when writing in English.

□Strongly disagree □Disagree □Neither Agree nor Disagree □Agree □Strongly agree

23. I am afraid of my English writing being chosen as a sample for discussion in class.

□Strongly disagree □Disagree □Neither Agree nor Disagree □Agree □Strongly agree

24. I am not afraid at all that my English writing would be rated as very poor.

□Strongly disagree □Disagree □Neither Agree nor Disagree □Agree □Strongly agree

25. Whenever possible, I would use English to write.

□Strongly disagree □Disagree □Neither Agree nor Disagree □Agree □Strongly agree
